# Study on Grain Refinement Mechanisms and Mechanical Properties of bi-Modal Ti-55511 Titanium Alloy during Hot Rolling

**DOI:** 10.3390/ma13153351

**Published:** 2020-07-28

**Authors:** Wei Chen, Xiaoyong Zhang, YongCheng Lin, Kechao Zhou

**Affiliations:** 1 State Key Laboratory of Powder Metallurgy, Central South University, Lu Mountain South Road, Changsha 410083, China; 143111047@csu.edu.cn (W.C.); zhoukechao@csu.edu.cn (K.Z.); 2 School of Mechanical and Electrical Engineering, Central South University, Changsha 410083, China; yclin@csu.edu.cn

**Keywords:** multi-pass hot rolling, bi-modal Ti-55511 alloy, mechanical properties, fragmentation of α phases, localized shear band

## Abstract

Multi-pass hot rolling was performed on bi-modal Ti-55511 alloy with 50% rolling reduction at 700 °C. Mechanical properties were evaluated by tensile test, and microstructure evolution was characterized by scanning electron microscopy (SEM) and transmission electron microscopy (TEM). The results show that the Ti-55511 alloy with bi-modal microstructure exhibits good strength and high ductility (1102 MPa, 21.7%). Comparatively, after 50% hot rolling, an enhanced strength and decreased ductility were obtained. The refinement of α phases leads to the increased tensile strength, while the fragmentation of the equiaxed α phase results in a decreased ductility. The fragmentation process of equiaxed α phases followed the sequence of: elongation of α phases → formation of grooves and localized shear bands → the final fragmentation accomplished via deepening grooves.

## 1. Introduction

Light weight is an urgent challenge in the field of aerospace structural components, marine industries, and implant materials. Titanium (Ti) alloys are widely and potentially useful, owing to its excellent properties, such as light weight, high strength, good fatigue resistance, and deep hardenability [1,2]. According to the phase component and stable phase, Ti-alloys can be divided into α, near α, α + β, near β, and β-Ti alloys. Near β-Ti alloys (Ti–5Al–5Mo–5V–1Cr–1Fe, Ti–5Al–5Mo–5V–3Cr, and Ti–10V–2Fe–3Al) have been widely utilized in aircraft structural materials for developing loading-bearing components [3]. In the past few decades, much research has been carried out to develop near β-Ti alloys with diverse microstructures, as an important developing direction of Ti-alloy with better overall properties. However, low plasticity is still a big challenge to the application of near β-Ti alloys, which is common in hexagonal close-packed (HCP) materials, such as Mg, Zr, and Ti alloys [4].

It is well known that the mechanical property is dependent on the microstructure morphology. As dual-phase Ti-alloys, the near β-Ti alloy is composed of a hexagonal close-packed (HCP) α phase and body-centered cubic (BCC) β matrix. Therefore, the mechanical property of the near β-Ti alloy is closely related to the microstructure morphologies of these two phases. The different morphologies can be obtained via various thermomechanical processing (TMP) routes. The common steps involved in the TMP routes are: (a) solution treatment; (b) deformation; (c) annealing or aging; and (d) recrystallization [5]. With various TMP routes, near β-Ti alloys can be lamellar, equiaxed, and bi-modal structures which permit a wide range of mechanical properties [6,7,8]. Compared with lamellar and equiaxed microstructures, the bi-modal microstructure processes better balance of strength and ductility, and it can be acquired via thermal deformation in the α+β field followed by heat treatment [6]. Meanwhile, the bi-modal near β-Ti alloy is still not a perfect candidate for high strength materials.

Grain refinement is a well-accepted method to improve combination properties of metals. According to the well-known Hall–Patch law, refining grain size can increase the yield stress of alloys. For example, for Ti–6Al–4V alloys, the mechanical strength increases with the decreasing grain size [9]. In recent years, a number of plastic deformation processes have been developed to refine the grain size of metallic alloys: (1) achieving grain refinement via severe plastic deformation (SPD) process; (2) precipitating nano-sized α particles by nucleating from pre-existing martensite phases [10,11,12]. Except for the α phase, the grain refinement of β matrix should also be addressed in Ti-alloys. In previous work, it was found that multi-pass cross-rolling can effectively refine β matrix better than unidirectional rolling [13,14]. Considering the crystal structures of α and β phases, the grain refinement mechanism should be different. For example, during hot rolling, the refinement mechanism of the α phase is continuous dynamic recrystallization (CDRX), while the main mechanism for the β phase is discontinuous dynamic recrystallization (DDRX). In addition, compared with the β phase, twinning is the main deformation mode owing to limited slip system. Chen et al. [15] demonstrated that dynamic recrystallization (DRX) process of lamellar α phases was accomplished by the following process: formation of sub-boundaries and high-angle α/α boundaries or shear bands across the α plates → formation of grooves at the α/β boundaries → deepening of grooves (penetration of β phase) → complete separation [15]. However, little information about the mechanism of equiaxed α phases can be found. Hence, it is very meaningful to investigate the grain-refinement mechanism of the equiaxed α phase.

Therefore, the present study aims to explore the grain refinement mechanisms of a bi-modal near β-Ti alloy during hot rolling. It also investigates the effects of hot rolling on the evolution of mechanical properties. For these purposes, the bi-modal Ti-55511 alloy was subjected to hot rolling at 700 °C. The microstructure characteristics of the alloys before and after hot rolling were analyzed by scanning electron microscopy (SEM) and transmission electron microscopy (TEM).

## 2. Materials and Methods

**Figure 1:**
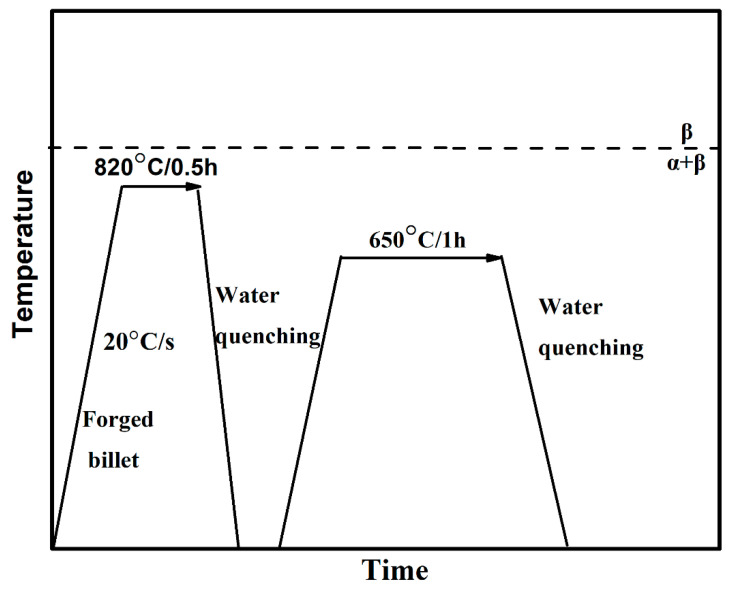
Schematic processing route for bi-modal microstructure.

The raw material utilized in the present research was a forged TC18 alloy billet (composition: Ti–5.16Al–4.92Mo–4.96V–1.10Cr–0.98Fe) provided by Xiangtou Goldsky Titanium Industry. The α + β → β transus temperature measured by metallographic method was approximately 875 ± 5 °C. Two cuboid samples (75 mm × 45 mm × 15 mm) were cut from the forged billet. Bi-modal microstructure was obtained by the heat treatment cycle as shown in Figure 1. After the heat treatment cycle, one of the cuboids was subjected to hot rolling with rolling reduction of up to 50% at 700 °C. During hot rolling, 5% rolling reduction was adopted in each pass.

Rectangular tensile samples with the dimension of 50 × 10 × 2 mm^3^ were machined out from the non-deformed and rolled sheets. The tensile tests were performed at room temperature using a CMT4204 mechanical testing machine (SUST, Zhuhai, China). Each test was repeated five times.

Microstructural characterization was performed by SEM (JSM-6360LV, Tokyo, Japan) and TEM (titan G2 60-300, FEI, Hillsboro, OR, USA). The SEM samples were prepared by grinding and subsequent light etching. The TEM specimens were compressed into thin foils (40–50 μm) and twin-jet (Denmar Struers A/S) electropolished (jet thinning) with a solution of 60% methanol, 35% butanol, and 5% perchloric acid.

## 3. Results and Discussion

**Figure 2:**
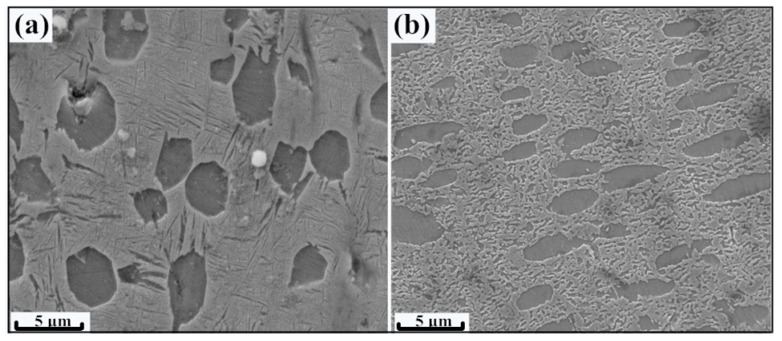
SEM morphologies of bi-modal Ti-55511 alloys (**a**) before and (**b**) after hot rolling.

Figure 2 shows the microstructures of the two bi-modal samples before and after 50% hot rolling (abbreviated as samples BM (bi-modal) and HR-BM (bi-modal after hot-rolling)). It can be seen that the bi-modal microstructure is mainly composed of equiaxed α phases (average grain size 3.5 μm), fine needle-like α phases and β phase matrix. After 50% hot rolling, the needle-like α phase was mostly fragmented into fine equiaxed nano-sized grains owing to DRX. For the equiaxed α phase, the grain was elongated along rolling direction, which is termed as the characteristic of dynamic recovery (DRV). One the other hand, the size of these grains (average 1.9 μm) is obviously smaller than that of sample BM, which the refined grains indicated the occurrence of dynamic recrystallization. It means that the DRX and DRV both occurred in the equiaxed α phase during 700 °C hot rolling.

### Mechanical Properties

**Figure 3:**
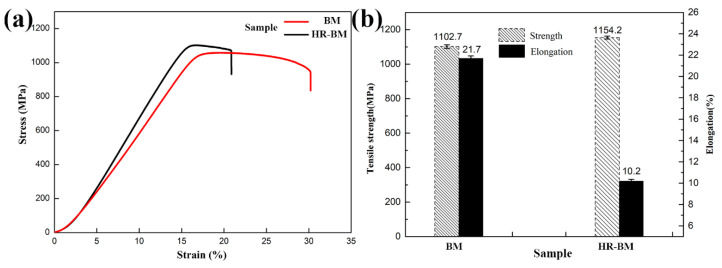
(**a**) Stress–strain curves, and (**b**) tensile strengths and elongations of samples BM and HR-BM.

Figure 3a shows the unidirectional tensile stress–strain of sample BM and HR-BM at room temperature. Sample HR-BM is stronger than sample BM, while sample BM is more plastic than sample HR-BM. The tensile strength and elongation of these two samples are shown in Figure 3b. The tensile strength and elongation are 1102.7 MPa and 21.7%, and 1154.2 MPa and 10.2% for BM and BM-HR, respectively. According to the morphologies of these two samples, the enhanced strength mainly results from the grain refinement of α phases. The variation of the ductilities of the samples is most likely related to the evolution of equiaxed α phases.

**Figure 4:**
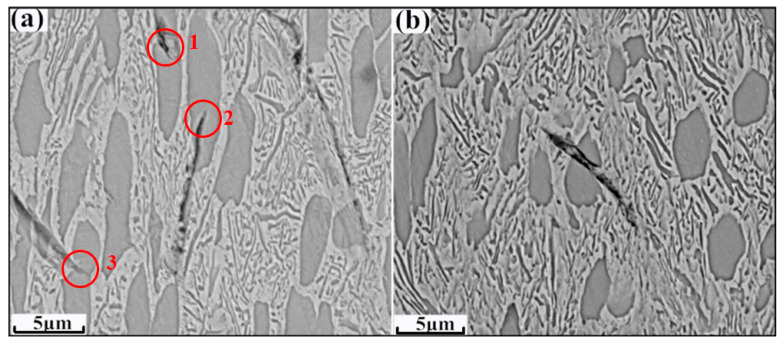
Profile-view SEM images close to the fracture surface of samples (**a**) BM (cracks inhibition in red circles) and (**b**) HR-BM.

To further elucidate the effect of the α morphologies on the plastic performance of samples BM and HR-BM, the profile-view SEM images close to the fracture surface of these two samples were investigated, as shown in Figure 4. After tensile loading, the equiaxed α grains were elongated along the loading direction in both samples. In addition, it is clear that the cracks both occurred in samples BM and HR-BM. As shown in Figure 4a, the cracks were mostly inhibited in the elongated α plates (in red circles 1, 2 and 3). In comparison, the crack propagated through the elongated α plate in Figure 4b. It should be mentioned that the size of elongated α plates in sample BM is bigger than that of sample HR-BM. Previous work demonstrated that the BCC β matrix is considerably softer than the HCP α phase at elevated temperature, which was attributed to more active slip systems in the β matrix than the α phase [16]. The initiation and propagation of cracks are closely related to the dislocation slip [17]. Therefore, the crack propagates in β matrix more easily, and namely, the α phase can effectively inhibit the crack propagation. On the other hand, the grain size has an obvious influence on the crack propagation resistance, and the increasing grain size can extend the crack length [14,15], and finally improves the ductility [18].

As mentioned above, the equiaxed α phase was generally elongated along the loading directions (rolling (Figure 2) or tensile directions (Figure 4)). Generally speaking, the large equiaxed α plates were considerably harder to refine than lamellar ones during unidirectional rolling [15]. It is well known that during high temperature deformation, the main grain refinement mechanism of the lamellar α phase was CDRX [17,18]. As depicted in Figure 2b, the elongated α phase is recognized as the characteristics of dynamic recovery (DRV) process. It can be noticed that, some large α grains in sample HR-BM are obviously smaller than that of sample BM. However, the studies of the grain refinement mechanism of equiaxed α phases are limited. In view of the aforementioned issue, it is of interest to study the grain refinement mechanism of the large α plates.

**Figure 5:**
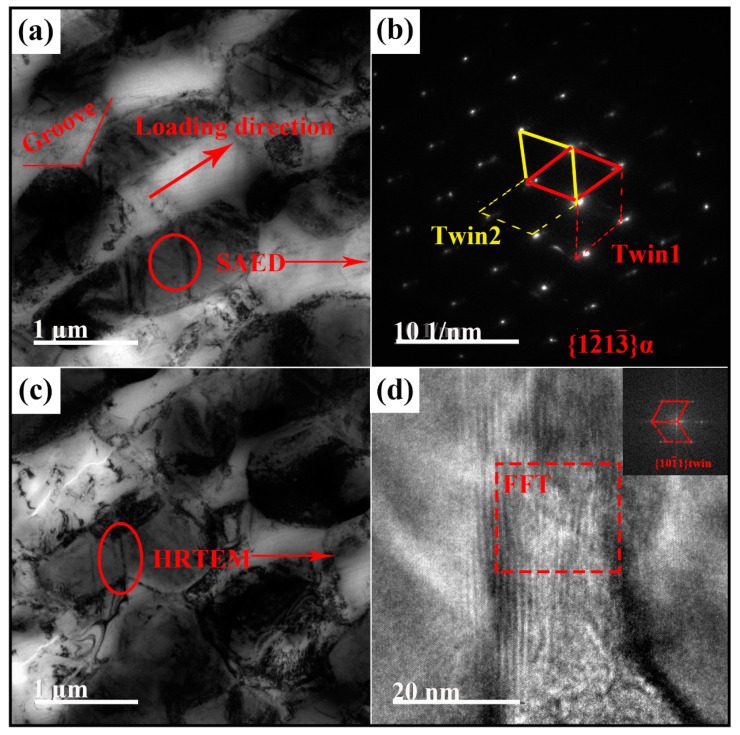
(**a**,**c**) TEM images of microstructural deformation characteristics of equiaxed α plates in sample HR-BM. (**b**) Selected-area diffraction pattern along the ${\left[1\bar{2}1\bar{3}\right]}_{\alpha }$ zone axis showing crossing twins identified as two twin variants of the $\left\{1\bar{1}01\right\}$ twinning system in the elongated α plate. (**d**) High-resolution TEM image of a localized shear band in the α particle in (**c**) and the corresponding fast Fourier transform (FFT) image verifying that the localized shear band is a $\left\{10\bar{1}1\right\}$ twin band.

More deformation features during hot deformation were further revealed by TEM imaging. Figure 5 shows the microstructures of sample HR-BM. After hot rolling, large α plates were elongated along loading direction, and grooves formed at the α/β interfaces (Figure 5a). Previous work demonstrated that the formation of the groove underlies the boundary splitting process which results in the spheroidization (breaking through) of α lamellas [19,20,21]. Furthermore, as depicted in Figure 5a, fine twins were observed in the elongated α plates. Analysis of the elected area electron diffraction (SAED) pattern (Figure 5b) verifies the presence of the $\left\{10\bar{1}1\right\}$ twining system, and furthermore, two twin variants of the $\left\{10\bar{1}1\right\}$ twinning system activated in these α plates. High-resolution spherical aberration-corrected TEM imaging was employed to view the atomistic structure of a triple-point junction near the groove (Figure 5c), as shown in Figure 5d. A localized shear band is clearly evident on the α side of the groove. The corresponding fast Fourier transform (FFT) pattern of the long band along the ${\left[1\bar{2}1\bar{3}\right]}_{\alpha }$ zone axis is inserted in the circled area of Figure 5d. It is found that the band is actually a $\left\{10\bar{1}1\right\}$ twin band. According to previous work [15,22,23], the localized shear band ties to the α/β interface and transversely forms across the α plate. The break-up of α plates or namely the β penetration accompanies when grooves deepen. Thus, in the present study, it can be implied that, for equiaxed α phases, the localized shear band, namely the twin band, forms across the elongated α phase, and the subsequent spheroidization process was accomplished by the grooves deepening.

## 4. Conclusions

In summary, the bi-modal Ti-55511 alloy was subjected to 50% hot rolling at 700 °C. The evolution of microstructure and tensile properties was investigated. The results show that the bi-modal microstructure processes a high ductility, because the large equiaxed α phase can effectively prohibit the propagation of cracks. After 50% hot rolling, the tensile strength increased owing to the grain refinement of α phases. After 50% hot rolling, the needle-like α phase fragmented into fine equiaxed nano-sized grains, while the equiaxed α phase elongated along the rolling direction. Meanwhile, the elongated α plate was fragmented into smaller ones in the subsequent deformation via deepening grooves, which is similar with the fragmentation process of the α lamellae.

## References

[1] Boyer R.R. (2013). An overview on the use of titanium in the aerospace industry. Mater. Sci. Eng. A.

[2] Banerjee D., Williams J.C. (2013). Perspectives on titanium science and technology. Acta Mater..

[3] Boyer R.R., Briggs R.D. (2013). The use of β titanium alloys in the aerospace industry. J. Mater. Eng. Perform..

[4] Yu Q., Shan Z., Li J., Huang X., Xiao L., Sun J., Ma E. (2010). Strong crystal size effect on deformation twinning. Nature.

[5] Jha J.S., Toppo S.P., Singh R., Tewari A., Mishra S.K. (2019). Flow stress constitutive relationship between lamellar and equiaxed microstructure during hot deformation of Ti–6Al–4V. J. Mater. Process. Technol..

[6] Chen W., Li C., Zhang X.Y., Chen C., Lin Y.C., Zhou K.C. (2019). Deformation-induced variations in microstructure evolution and mechanical properties of bi-modal Ti-55511 titanium alloy. J. Alloys Compd..

[7] Semiatin S.L., Seetharaman V., Weiss I. (1997). The thermomechanical processing of α/β titanium alloys. JOM.

[8] Lütjering G., Williams J.C. (2007). Engineering Materials and Processes-Titanium.

[9] Chen W., Chen C., Zi X.H., Cheng X.F., Zhang X.Y., Lin Y.-C., Zhou K.C. (2018). Controlling the microstructure and mechanical properties of a metastable β titanium alloy by selective laser melting. Mater. Sci. Eng. A.

[10] Yu H.L., Yan M., Li J.T., Godbole A., Lu C., Tieu K., Li H.J., Kong C. (2018). Mechanical properties and microstructure of a Ti-6Al-4V alloy subjected to cold rolling, asymmetric rolling and asymmetric cryorolling. Mater. Sci. Eng. A.

[11] Zhao X.C., Yang X.R., Liu X.Y., Wang X.Y., Langdon T.G. (2010). The processing of pure titanium through multiple passes of ECAP at room temperature. Mater. Sci. Eng. A.

[12] Chen S.F., Song H.W., Zhang S.H., Cheng M., Lee M.G. (2015). Equal channel angular bending as a new severe plastic deformation process: Application to thin Mg-3Al-1Zn sheet. Mater. Lett..

[13] Sun W.T., Qiao X.G., Zheng M.Y., He Y., Hu N., Xu C., Gao N., Starink M.G. (2018). Exceptional grain refinement in a Mg alloy during high pressure torsion due to rare earth containing nanosized precipitates. Mater. Sci. Eng. A.

[14] Chen W., Lv Y.P., Wang H.D., Zhang X.Y., Chen C., Lin Y.C., Zhou K.C. (2019). On the $\left\{1\bar{1}01\right\}$ twin-accommodated mechanisms in equiaxed near β-Ti alloys operating by unidirectional and cross rolling. Mater. Sci. Eng. A.

[15] Chen W., Wang H.D., Lin Y.C., Zhang X.Y., Chen C., Lv Y.P., Zhou K.C. (2020). The dynamic responses of lamellar and equiaxed near β-Ti alloys subjected to multi-pass cross rolling. J. Mater. Sci. Technol..

[16] Chen W., Lv Y.P., Zhang X.Y., Chen C., Lin Y.C., Zhou K.C. (2019). Comparing the evolution and deformation mechanisms of lamellar and equiaxed microstructures in near β-Ti alloys during hot deformation. Mater. Sci. Eng. A.

[17] Ritchie R.O. (2011). The conflicts between strength and toughness. Nat. Mater..

[18] Wu C., Zhan M. (2019). Microstructural evolution, mechanical properties and fracture toughness of near b titanium alloy during different solution plus aging heat treatments. J. Alloys Compd..

[19] Shen J.Y., Sun Y., Ning Y.Q., Yu H., Yao Z.K., Hu L.X. (2019). Superplasticity induced by the competitive DRX between BCC beta and HCP alpha in Ti-4Al-3V-2Mo-2Fe alloy. Mater. Charact..

[20] Liu H., Fujii H. (2018). Microstructural and mechanical properties of a beta-type titanium alloy joint fabricated by friction stir welding. Mater. Sci. Eng. A.

[21] Zhang W.J., Liu H.H., Ding H., Fujii H. (2019). Grain refinement and superplastic flow in friction stir processed Ti-15V-3Cr-3Sn-3Al alloy. J. Alloys Compd..

[22] Jia C.L., Kou H.C., Chen N.N., Liu S.B., Fan J.K., Tang B., Li J.S. (2019). Stress relaxation induced spheroidization of the lamellar a phase in Ti-7333 alloy. J. Alloys Compd..

[23] Weiss I., Froes F.H., Eylon D., Welsch G.E. (1986). Modification of alpha morphology in Ti-6Al-4V by thermomechanical processing. Mater. Trans. A.

